# Single Layer Centrifugation Can Be Scaled-Up Further to Process up to 150 mL Semen

**DOI:** 10.5402/2011/183412

**Published:** 2012-01-31

**Authors:** J. M. Morrell, M. van Wienen, M. Wallgren

**Affiliations:** ^1^Division of Reproduction, Department of Clinical Sciences, Swedish University of Agricultural Sciences (SLU), P.O. Box 7054, 75007 Uppsala, Sweden; ^2^Faculty of Veterinary Medicine, Utrecht University, 3584 CL Utrecht, The Netherlands; ^3^Quality Genetics, 24292 Hörby, Sweden

## Abstract

Single-Layer centrifugation has been used to improve the quality of sperm samples in several species. However, where stallion or boar semen is to be used for AI, larger volumes of semen have to be processed than for other species, thus limiting the effectiveness of the original technique. The objective of the present study was to scale up the SLC method for both stallion and boar semen. Stallion semen could be processed in 100 mL glass tubes without a loss of sperm quality, and similarly, boar semen could be processed in 200 mL and 500 mL tubes without losing sperm quality. The results of these preliminary studies are encouraging, and larger trials are underway to evaluate using these methods in the field.

## 1. Introduction

Single Layer centrifugation (SLC) through a colloid has been advocated as a biomimetic method for improving sperm quality in artificial insemination (AI) doses, since it selects morphologically normal spermatozoa with intact membranes and good chromatin integrity in a similar manner to the selection processes occurring in the female reproductive tract [1]. Capacitation and the acrosome reaction are not affected by SLC in stallion spermatozoa [2]. In addition to selecting the most robust spermatozoa, SLC also removes them from seminal plasma [3]. Since morphology and chromatin integrity have been linked to pregnancy rates in mares following AI [4], it has been postulated that the improvement in sperm quality seen following SLC could be reflected in improved pregnancy rates following (AI) if other factors (such as timing of insemination relative to ovulation and the skill of the inseminator) are optimal. Species-specific formulations of Androcoll have been developed for stallion [5], boar [6], bull [7], dog [8], and cat [9].

**Figure 1:**
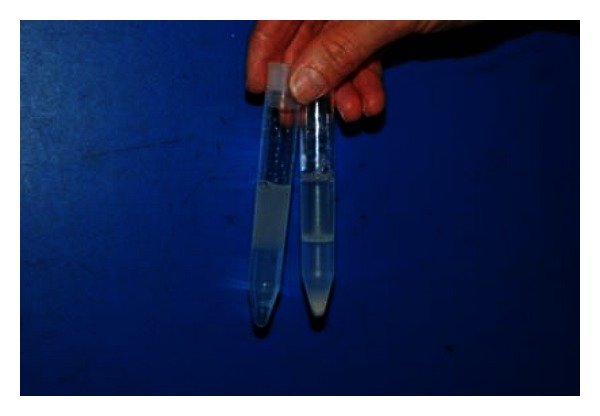
SLC-4.5. The tube on the left shows the SLC-4.5 prior to centrifugation, with 4.5 mL extended boar semen on top of 4.0 mL Androcoll-P. The tube on the right shows the preparation after centrifugation with the sperm pellet clearly visible in the bottom of the tube. Note the white line marking the interface between semen and colloid after centrifugation. This consists of spermatozoa that have not been able to pass into the colloid because of poor motility, abnormal morphology, or damaged chromatin.

**Figure 2:**
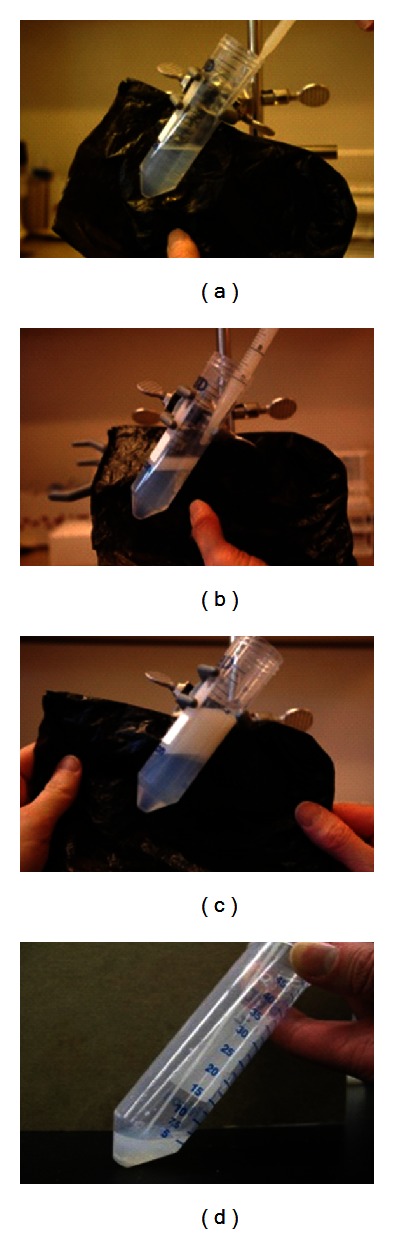
SLC-15. The various steps in the preparation of SLC-15 with stallion semen. (a) The first 1.5 mL (the most sensitive) is added from a Pasteur pipette to avoid disrupting the colloid surface and create a sharp interface; (b) The remaining semen is added from a disposable pipette; (c) All the semen has been layered on the colloid (note that the interface between semen and colloid is clearly delineated); (d) After centrifugation, the supernatant is removed by aspiration and the sperm pellet is aspirated using a clean pipette.

A major obstacle to the use of SLC to process stallion or boar spermatozoa for (AI) is the large volume of the ejaculate and the relatively high sperm number in the insemination dose [10]. Therefore, it would not be practical to use the original SLC method in the field to prepare spermatozoa for AI, since approximately 100–300 tubes each processing 1.5 mL extended semen would be needed to produce the requisite number of spermatozoa for equine or porcine AI. Clearly, these numbers are impractical. Even scaling up to 4.5 mL extended semen in 12 mL tubes is only feasible for a small number of ejaculates (Figure 1) [11]. The SLC method has been scaled-up successfully with stallion semen from 12 mL tubes into 50 mL tubes (Figure 2) [11], whereas the method for boar semen has been scaled-up into 50 mL and 100 mL tubes without apparent loss of sperm quality [12, 13]. Morrell et al. [11] also reported a method for SLC in 200 mL tubes for stallion semen although these tubes do not fit the types of centrifuge commonly found on studs. However, in some cases (particularly for boar semen), it would be convenient to be able to process even larger volumes of semen per tube to avoid using more than one centrifuge or processing tubes in more than one centrifuge cycle.

The purpose of the preliminary studies reported here was to investigate the possibility of scaling-up SLC with species-specific formulations of Androcoll (Androcoll-E or Androcoll-P, where the suffix denotes equine or porcine) to process larger volumes of stallion and boar semen than hitherto attempted. The different scale-up sizes are referred to henceforth by the volume of extended semen processed; for example, SLC-15 refers to processing 15 mL extended semen.

## 2. Materials and Methods

### 2.1. Experiment  1: Scale-Up into 100 mL Glass Tubes (Stallion Semen)

Warmblood stallions (4) of breeding age (7 to 23 years old) and known fertility were housed under standard husbandry conditions at a commercial stud in Sweden. Semen was collected during the month preceding the breeding season at the Division of Reproduction, Swedish University of Agricultural Sciences (SLU), Uppsala, Sweden, as part of a teaching demonstration. The stallions were allowed to mount a phantom and ejaculated into a warmed artificial vagina (Colorado type), the semen being collected into a warm plastic bottle with an in-line filter to capture gel. The semen was immediately extended 1 : 1 with warm (35°C) INRA96 semen extender (IMV Technologies, l'Aigle, France). After measuring the sperm concentration with the Nucleocounter SP-100 [13], samples with a sperm concentration higher than 100 × 10^6^/mL were adjusted down to this level, which has previously been determined to be optimal for SLC [15]. Aliquots (4.5 mL or 25 mL) of extended semen were carefully pipetted on top of Androcoll-E in 12 mL plastic centrifuge tubes or 100 mL glass conical tubes, respectively, followed by centrifugation at 300 g for 20 min. After aspirating the supernatant (consisting of extender, seminal plasma, dead spermatozoa at the interface with the colloid, and most of the colloid) using a water pump, the sperm pellets were harvested into fresh extender, and the sperm concentration of the resulting suspension was adjusted as appropriate (approximately 50 × 10^6^/mL) for computer-assisted sperm analysis (CASA). Immediately after SLC, aliquots of each sample were collected for subsequent morphological examination [4, 16]. Sperm motility was measured immediately with the Cell Motion Analyser (MTM Medical Technologies Montreux, Switzerland) as described previously [17] and again after storage of the sperm samples for 24 and 48 h at 6°C. Mean values for motility were compared using the Generalised Linear Model mixed procedure after arcsine transformation of the data, with repeated measures over time. For morphology, only the Generalised Linear Model was used. Differences were considered to be significant at *P* < 0.05.

### 2.2. Experiment  2: Scale-Up into 200 mL Tubes (Boar Semen)

Four boars aged 3-4 years, (Swedish Landrace, Swedish Yorkshire and two Norwegian Landrace) were housed at the Division of Reproduction, Swedish University of Agricultural Sciences, Uppsala, Sweden, under standard husbandry conditions [18]. The sperm-rich fraction from each of 12 ejaculates (three per boar) was collected using the gloved-hand technique and was immediately extended 1 : 1 (v/v) in warm (35°C) Beltsville Thawing Solution (BTS) [19] without antibiotics [5]. Sperm concentration, measured using the Nucleocounter SP-100 (Chemometec, Denmark) [20], was adjusted to approximately 100 × 10^6^ spermatozoa/mL. Aliquots of extended semen (15 mL) were layered on top of 15 mL Androcoll-P Large in 50 mL tubes (SLC-15) and 60 mL extended semen was layered on top of 50 mL Androcoll-P (S) in 200 mL centrifuge tubes (SLC-60). After centrifugation at 300 g for 20 min, the sperm pellets were resuspended in BTS containing bSA (1.25 mg/mL) and sperm quality (motility, morphology, and viability) was determined as previously described [13]. Mean values were compared by ANOVA. Another sperm sample, processed by SLC-15 and SLC-60, was frozen by a modified simplified method [20].

### 2.3. Experiment  3: Scale-Up into 500 mL Tubes (Boar Semen)

**Figure 3:**
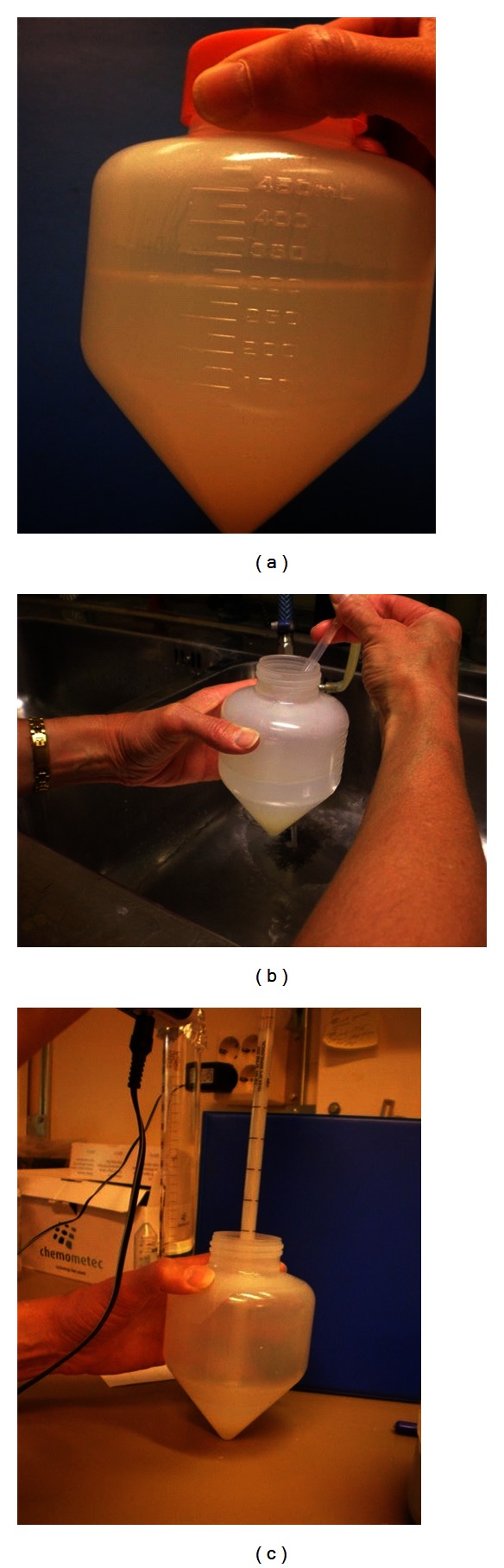
SLC-150. Notes: (a) After centrifugation, the different layers can be seen, comprising 150 mL seminal plasma and extender on top of 150 mL colloid, with the sperm pellet in the bottom of the tube; (b) Removing the supernatant after centrifugation using a water pump; (c) Aspirating the sperm pellet.

Aliquots (150 mL) of boar semen from one of the boars used for experiment 2, extended to 100 × 10^6^ spermatozoa/mL, were prepared by (i) SLC on top of 150 mL Androcoll-P (Large) in a 500-mL tube (SLC-150) and (ii) by simple centrifugation (sperm “washing”). After centrifuging at 300 g for 20 min, the supernatant was removed, and the sperm pellets were resuspended in Beltsville Thawing Solution (BTS) with added bovine serum albumin (5 mg/mL). In another experiment, aliquots of semen were prepared by SLC-150 or by SLC-15 (150 Androcoll-P (S) and 15 mL Androcoll-P Large, resp.) (Figure 2). Sperm motility in uncentrifuged control samples and in the SLC-selected samples was measured by computer-assisted motility analysis using the SpermVision (Minitube, Tiefenbach, Germany) both on the day of preparation and after seven days storage at 16°C–18°C in a Climate Box (Unitron, Denmark). Further aliquots of boar semen were prepared by SLC-150 on two subsequent occasions, with motility being measured on day 0 and day 7, as before (Figure 3).

## 3. Results

A summary of the volumes of colloid and semen and the proposed nomenclature for the various techniques is given in Table 1.

### 3.1. Experiment  1

**Figure 4:**
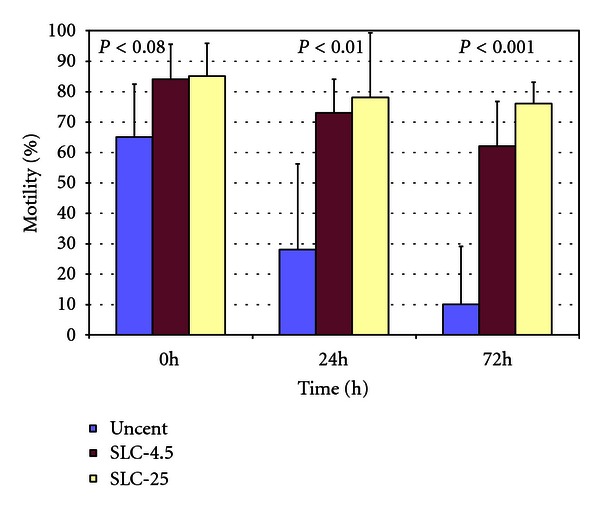
Progressive motility in uncentrifuged, SLC-4.5, and SLC-25 sperm samples (*n* = 4) during cold storage at 6°C for up to 72 h. Note: stallion spermatozoa were prepared by SLC-4.5 and SLC-25 (4.5 mL extended semen on top of 4 mL Androcoll-E in a 12-mL centrifuge tube, and 25 mL extended semen on top of 20 mL Androcoll-E in a 100 mL conical glass centrifuge tube, resp.); uncent = uncentrifuged control.

Progressive motility (PM) in the different samples is shown in Figure 4. There was a trend towards significance between the SLC samples (Figure 4) and the corresponding unprocessed controls (*P* < 0.08) at 0 h although the difference was significant at 24 h (*P* < 0.01) and at 72 h (*P* < 0.001). There were no differences, however, between the SLC-4.5 and SLC-25 treatments at any time points. The proportion of spermatozoa with normal morphology was statistically different for SLC-4.5 versus uncentrifuged (SLC-4.5 82 ± 11%, uncentrifuged 64.5 ± 15%; *P* < 0.01) but not for SLC-25 versus uncentrifuged (SLC-25 73 ± 17%, uncentrifuged 64.5 ± 15%; not significant).

### 3.2. Experiment  2

Mean values (±SD) for PM, normal morphology, and membrane integrity of the boar sperm samples are shown in Table 2. There were no significant differences between the results for SLC-15 and SLC-60. After freezing, progressive motilities were 35% and 54% for SLC-15 and SLC-60, respectively.

### 3.3. Experiment  3

**Figure 5:**
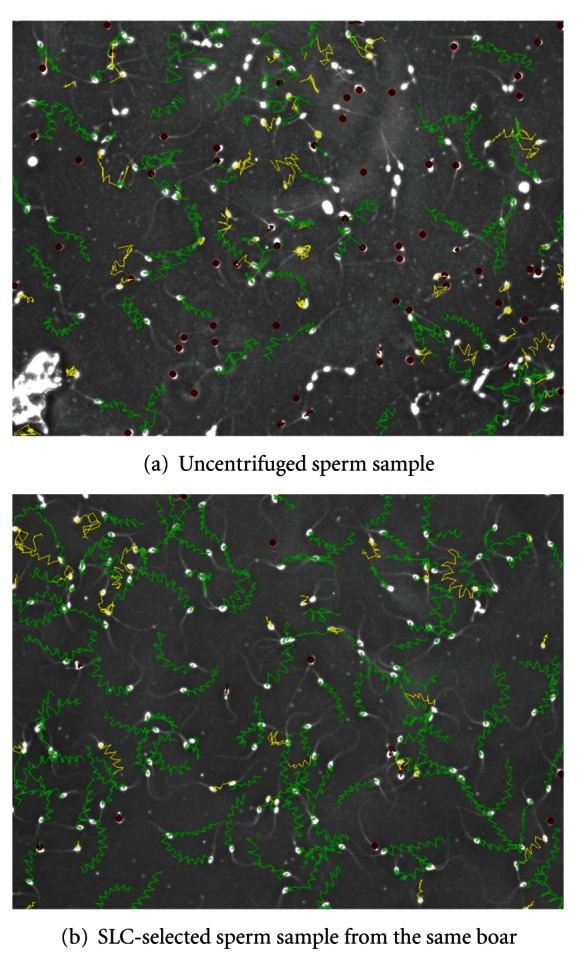
Sperm tracks obtained during motility analysis of uncentrifuged and SLC-selected boar sperm samples using the SpermVision motility analyzer. Note: red dots indicate immotile spermatozoa, green tracks indicate progressively motile spermatozoa, and yellow tracks indicate locally motile spermatozoa.

Sperm motility in the sperm samples was as follows: immediately SLC-150 82%, wash 61%, and control 68% and after storage for 7 days at 16°C–18°C (SLC 75%, wash 55%, and control 70%). The yield from SLC-150 was 80%–86%. The patterns of sperm motility are shown in Figure 5; progressive motility at time 0: SLC-15 92%, SLC-150 87%, control 66% and after 7 days: SLC-15 69%, SLC-150 91%, and control 64%. When combined with the results for the other two SLC-150 preparations, the mean motilities for the uncentrifuged control and SLC-150 sperm samples on Day 7 were 68 ± 14% and 87 ± 4%, respectively.

## 4. Discussion

The purpose of these preliminary experiments was to investigate whether the SLC-technique could be scaled up to facilitate processing larger volumes of extended stallion and boar semen than hitherto routinely used. The volumes of Androcoll chosen gave approximately the same height of the colloid column in the different centrifuge tubes, since our previous work had suggested that this is an important factor in the success of the technique [11].

In the first experiment, parameters of stallion sperm quality were comparable in the large-scale SLC-samples and smaller volume SLC, suggesting that it is possible to scale up the technique for stallion semen in 100 mL glass tubes without compromising semen quality. These results are in agreement with previous results, which showed that SLC-25 (in 100 mL tubes) was possible for boar semen [13]. Furthermore, AI has been carried out using SLC-25 sperm samples in five mares, of which three conceived (J. M. Morrell, unpublished data), indicating that spermatozoa retain their functional capacity as well as their *in vitro* sperm quality after scaled-up SLC.

The preliminary results of the boar semen experiments reported here show that scaling up the technique for use in 200 mL and 500 mL tubes is possible. The results resemble those obtained previously for SLC-60 (in 200 mL tubes) with stallion semen [11]. Since similar results were obtained for both species with each SLC scale-up, it can be assumed that these results will hold for other species as well and also for SLC-150 with stallion semen. Boar sperm survival after SLC-150 was particularly noteworthy, since PM was 75% after 7-day storage in extender without antibiotics. It would be desirable to try to reduce antibiotic usage whilst still maintaining the biosecurity of semen doses in an attempt to limit the spread of antibiotic resistance [21].

SLC-60 selected spermatozoa were used successfully for freezing, showing higher postthaw progressive motility than SLC-15. This result may be due to a higher sperm concentration in the sperm pellet. If this is the case, it should also be possible to improve the freezing of SLC-15 selected spermatozoa by centrifuging the sperm suspension one more time to concentrate the sperm pellet before resuspension in the cryoprotectant. Alternatively, use of the SLC-150 would allow all of the extended sperm-rich fraction (SRF) of the boar ejaculate to be processed in three or four 500 mL tubes in one centrifugation cycle. This method could be of interest for the boar semen industry, and a larger trial is currently underway to assess the usefulness of the method.

The SLC technique is more convenient and less time consuming than density gradient centrifugation (DGC), which has previously been used occasionally to prepare animal semen [9]. SLC is also more versatile than DGC, allowing the method to be scaled up into larger tubes, which is not practical for DGC [21] because of the time involved in layering several colloids, each of a different density. Once the operator becomes familiar with the techniques, the layering of the extended semen on top of the colloid for SLC and the subsequent harvesting of the sperm pellet are not time consuming, such that a whole boar ejaculate can be processed in approximately 40 min by SLC-150, including the 20 min centrifugation.

In conclusion, the SLC technique can be scaled up to process 25 mL stallion semen or 60 mL and 150 mL boar semen without loss of sperm quality.

## Figures and Tables

**Table 1:** Summary of the volumes of colloid and extended semen*, centrifuge tubes, and centrifuges used in these experiments (modified from [14]).

Species	Tube size	Volume of colloid (mL)	Volume of extended semen* (mL)	Name of technique	Centrifuge
Stallion/boar	12 mL	4.0	4.5	SLC-4.5	1
Stallion/boar	50 mL	15	15–18	SLC-15	1
Stallion/boar	100 mL	20	25	SLC-25	2
Stallion/boar	200 mL	60	60	SLC-60	3
Boar**	500 mL	150	150	SLC-150	1

^∗^Extended to give a concentration of approximately 100 × 10^6^ spermatozoa/mL.

^∗∗^this technique has not been attempted yet with stallion semen but is theoretically possible.

Centrifuge 1: for example: Hereaus Multifuge, Kendro, Osterode, Germany;

Centrifuge 2: for example: Sigma Laboratory Centrifuge, Osterode, Germany;

Centrifuge 3: for example: Centra MP 4R, International Equipment Company, MA, USA.

**Table 2:** Mean values (±SD) of parameters of sperm quality in SLC-selected (SLC-15 and SLC-60) boar sperm samples (*n* = 12).

Sample	Progressive motility (%)	Normal morphology (%)	Membrane integrity (%)
SLC-15	87 ± 11	94 ± 5	94 ± 3
SLC-60	87 ± 11	87 ± 3	95 ± 3

Note: SLC-15 and SLC-60 refer to 15 mL extended semen on top of 15 mL Androcoll-P (Large) and 60 mL extended semen on top of 60 mL Androcoll-P, respectively.
